# High-throughput metagenome analysis of the *Sarcoptes scabiei* internal microbiota and *in-situ* identification of intestinal *Streptomyces sp.*

**DOI:** 10.1038/s41598-019-47892-0

**Published:** 2019-08-13

**Authors:** Pearl M. Swe, Martha Zakrzewski, Rebecca Waddell, Kadaba S. Sriprakash, Katja Fischer

**Affiliations:** 1 Infectious Diseases Program, Cell and Molecular Biology Department, QIMR Berghofer Medical Research Institute, Brisbane, Australia; 2 Medical Genomics Program, Genetics & Computational Biology Department, QIMR Berghofer Medical Research Institute, Brisbane, Australia

**Keywords:** Parasite biology, Metagenomics

## Abstract

Multiple parasitic arthropods of medical importance depend on symbiotic bacteria. While the link between scabies and secondary bacterial infections causing post infective complications of Group A streptococcal and staphylococcal pyoderma is increasingly recognized, very little is known about the microbiota of *Sarcoptes scabiei*. Here we analyze adult female mite and egg metagenome datasets. The majority of adult mite bacterial reads matched with Enterobacteriaceae (phylum Proteobacteria), followed by Corynebacteriaceae (phylum Actinobacteria). *Klebsiella* was the most dominant genus (78%) and *Corynebacterium* constituted 9% of the assigned sequences. Scabies mite eggs had a more diverse microbial composition with sequences from Proteobacteria being the most dominant (75%), while Actinobacteria, Bacteroidetes and Firmicutes accounted for 23% of the egg microbiome sequences. DNA sequences of a potential endosymbiont, namely *Streptomyces*, were identified in the metagenome sequence data of both life stages. The presence of *Streptomyces* was confirmed by conventional PCR. Digital droplet PCR indicated higher *Streptomyces* numbers in adult mites compared to eggs. *Streptomyces* were localized histologically in the scabies mite gut and faecal pellets by Fluorescent *In Situ* Hybridization (FISH). *Streptomyces* may have essential symbiotic roles in the scabies parasite intestinal system requiring further investigation.

## Introduction

The mite *Sarcoptes scabiei* is the causative agent of the highly pruritic and contagious skin disease ‘scabies’ in humans and ‘mange’ in animals. Mechanical infringement of the intact skin resulting from the burrowing action of the mite into the epidermis and scratching by the host in response to itching provides access for opportunistic bacteria. The mite produces several classes of proteins with anti-complement and anti-inflammatory activities to protect itself from the onslaught of the host immune defence^1–3^. These proteins are released with the mite feces into the epidermal burrows and have been proposed to indirectly promote secondary bacterial infections, as they supress the local host immune defence^4,5^. Thus, they may contribute to rapid changes seen in the host skin microbiota in scabies lesions^6,7^. Scabies is now recognized as a risk factor for skin infections caused by *Staphylococcus aureus* and *Streptococcus pyogenes* in humans^8–13^. A considerable portion of scabies-associated secondary infections are thought to lead to serious downstream complications in the form of life-threatening *S*. *aureus* bacteraemia or severe post-streptococcal sequelae such as glomerulonephritis, rheumatic fever and/or rheumatic heart disease, amounting to a substantial morbidity and mortality^10,14–18^.

Parasitic arthropods have been reported to act as vectors for pathogenic bacteria. For example, the tick *Ixodes sp*. is a vector for *Borrelia burgdorferi* causing Lyme’s disease^19^ and *Dermacentor andersoni* is a vector for *Francisella tularensis* causing Tularemia^20^. It is hypothetically possible that the scabies mite may act as a carrier of pathogenic bacteria, as these have been localized in the mite feces^21^; however direct transmission of bacteria by mites between hosts has not yet been demonstrated.

In addition to pathogens, the microbiota of arthropods often include symbiotic bacteria beneficial to the arthropod host in providing, for example, specific essential nutrients that the host is unable to synthesize (reviewed in^22^) or may have a role in digestion of food^23,24^ or in protecting their hosts from toxicants^25^. Disruption or removal of the internal microbiota and endosymbionts in arthropods has been reported to reduce survival, fecundity and growth of arthropods^26–28^ and has led to the concept to control arthropod-borne disease by controlling their symbionts (reviewed in^29^).

Here we begin to explore the bacteria associated with the scabies parasite as a basis for dissecting their role in mite biology and pathogenicity. Of major importance for this research was unconstrained access to all developmental stages of *S*. *scabiei*. In the absence of an *in vitro* culture for scabies mites, an experimental porcine scabies model^30^ allowed a systematic approach, that would not have been possible if attempting to source the parasites from human patients. Human and porcine hosts are infected by the same species (*S*. *scabiei*). Although some physiological differences may determine host preferences, no unequivocal morphological differences exist between the two *S*. *scabiei* biovars^31^. Immunological cross reactivity has been demonstrated for multiple mite proteins (reviewed in^32^) and recent mitochondrial genome sequencing data indicate that some porcine and human biovars are genetically very closely related^33^. While we expect differences between the porcine and human mite internal microbiota at species level, symbiotic relationships could be conserved across the *S*. *scabiei* biovars, as their symbiotic function may place selection pressure on maintaing them. Careful extrapolation of principle findings from the porcine into the human context may be feasible.

We employed metagenome sequencing to elucidate the internal microbial composition in adult female mites and eggs. We identified *Streptomyces sp*. as a persistent component of the internal microbiota. As Streptomyces are known to be endosymbionts in other arthropods^34^, we investigated this possibility by digital droplet PCR (ddPCR) and Fluorescent *In Situ* hybridization (FISH). Although our results do not prove that *Streptomyces* are symbionts of *Sarcoptes scabiei*, their persistent presence in the mite intestinal system suggests that the bacterium may offer a survival advantage to the mites.

## Results

### 0.65% Sodium hypochlorite solution removed external DNA from the scabies eggs

**Figure 1:**
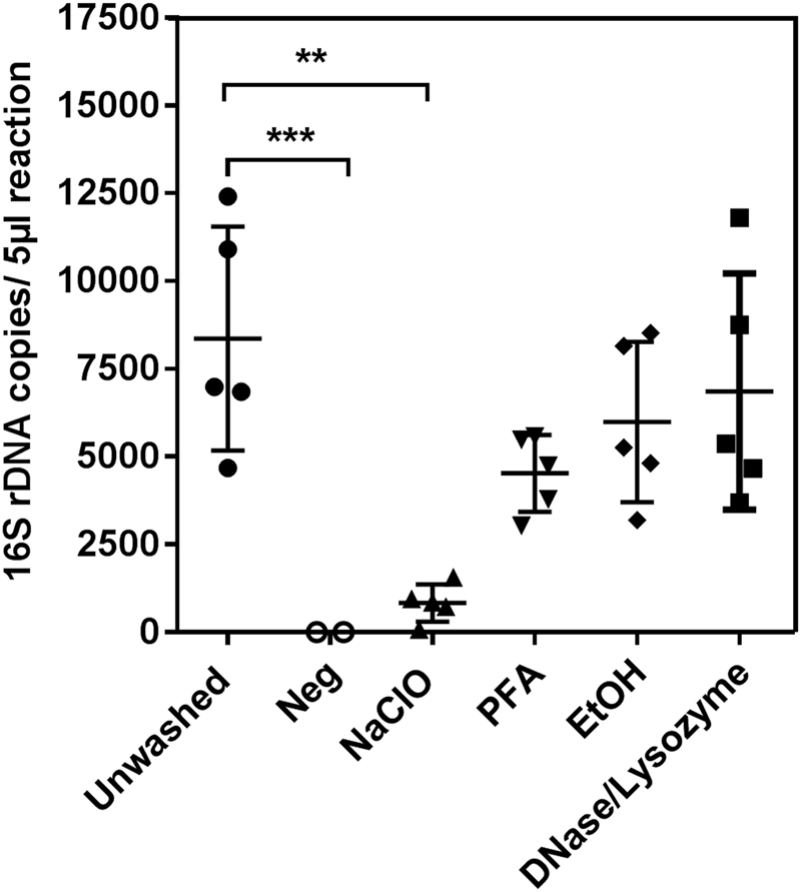
Comparison of cleaning procedures of scabies eggs. Concentrations of 16S rDNA amplicon copy numbers generated from scabies egg samples treated with four different cleaning solutions. Statistically significant differences between treated samples and compared to unwashed control are indicated by *. Statistical differences were determined using 1-way ANOVA, Dunnett’s multiple comparisons test (**p ≤ 0.005) and (***p ≤ 0.0005).

Using standard curve and Cp values generated through qPCR experiments, the absolute quantity of the 16S rDNA copies of bacteria in the samples after washing procedures (one to four) compared to unwashed samples was calculated. Only eggs treated with Sodium Hypochlorite solution showed significantly lower 16S rDNA copy numbers compared to unwashed eggs (p = 0.0014) and negative control (p = 0.0002), while copy numbers determined for Ethanol, PFA and DNase/Lysozyme-treated eggs were not significantly different from unwashed eggs (Figure 1). This indicated that the washing procedure one was most stringent and accordingly, for all following experiments, eggs and mites were washed with 0.65% Sodium hypochlorite solution to remove external bacterial DNA.

### Metagenome analysis of the microbiota of scabies mites and eggs

Illumina sequencing of genomic DNA prepared from washed adult mites or eggs resulted in 15,278,709 paired-end (PE) reads for the adult female metagenome and 8,160,557 PE reads for the egg metagenome, both with an average read length of 151 bp. Adapter and low quality reads were removed, resulting in 14,808,054 and 7,934,518 PE high quality reads in the adult female and egg datasets respectively. Reads were aligned against the published scabies mite and pig draft genomes, cleared of chimera sequences, resulting in 29,663 (0.2%) and 8,036 (0.09%) unmapped PE reads, representing the adult female and egg microbiomes respectively. In total, 81.1% and 38.2% of the unmapped PE reads from the adult female and egg microbiome data respectively were assigned to a bacterial taxon using the software tool Kraken.

**Figure 2:**
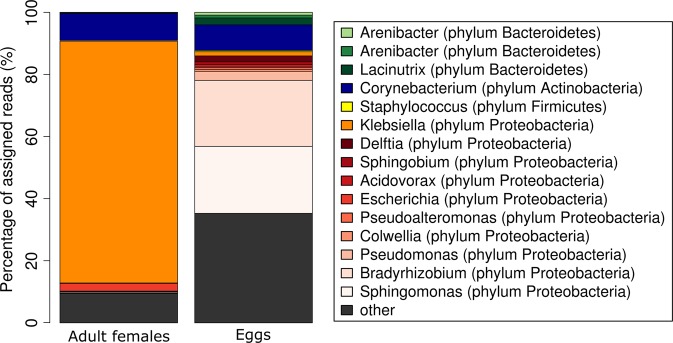
Barchart showing the relative abundances of bacteria in the metagenomes of adult female scabies mites and scabies eggs. Only the top 10 genera in each sample are listed. The remaining genera including *Streptomyces* are categorized to “others”. For more detail see S2_Fig. 1. Sequence data were annotated using Kraken and visualized using the software package R.

The phylum Proteobacteria dominated the adult female microbiome with 89% of all taxonomically assigned reads, followed by Actinobacteria with 9%. Within the phylum Proteobacteria, *Klebsiella* was the most abundant genus in the adult female microbiome accounting for 78% of all assigned reads (Fig. 2, S1_Tables 1 and 2, S2_Fig. 1). *Corynebacterium* (9% of all assigned reads) was the major taxon within the phylum Actinobacteria.

Compared to the adult female scabies mites, the egg microbiome showed a more heterogeneous composition (Fig. 2, S1_Tables 1 and 2, S2_Fig. 1). The phylum Proteobacteria was dominating with 75% of all assigned sequences. Within Proteobacteria, *Bradyrhizobium* and *Sphingomonas* were the most abundant taxa. The phyla Actinobacteria, Bacteroidetes, and Firmicutes accounted for 23% of the assigned egg microbiome reads with *Corynebacterium*, *Lacinutrix*, and *Staphylococc*us as well as *Streptococcus* being the most abundant genera. The genera *Staphylococcus* and *Streptococcus*, among which multiple species are opportunistic pathogens, were predicted in both the adult female and the egg microbiome read datasets when using Kraken and BLAST.

### Evidence of *Streptomyces* in the adult female mite and egg metagenomes

The genus *Streptomyces* (phylum Actinobacteria), which frequently plays symbiotic roles in arthropods, was predicted through the Kraken analysis in both the egg (0.7% of all assigned reads, 23 reads) and adult (0.04%, 9 reads) metagenomes. Conventional PCR confirmed the presence of *Streptomyces sp*. (data not shown). PCR detection for Eubacteria and *K*. *pneumoniae* was included as a positive control. PCR amplification products were sequenced using *Streptomyces* specific primers and sequences were confirmed as *Streptomyces sp*. in BLASTn analysis (Table 1).

**Table 1:** BLASTn analysis confirming the presence of *Streptomyces* in the adult female mite and egg metagenomes.

*Streptomyces mirabilis* strain B432
*Streptomyces galbus* strain CBT BW2
*Streptomyces cellulosae* strain VJDS
*Streptomyces rimosus* strain JA42
*Streptomyces flavofungini* strain BB1
*Streptomyces griseoaurantiacus* strain BB9
*Streptomyces albidoflavus* strain DT-A40
*Streptomyces parvus* strain 259-8
*Streptomyces macrosporeus* strain 1061
*Streptomyces humidus* strain FMA160
*Streptomyces albogriseolus* strain HBUM83454
*Streptomyces cacaoi* subsp. *cacaoi* strain AMJKG-M20
*Streptomyces violaceoruber* strain BCX44
*Streptomyces canus* strain LuP47B
*Streptomyces bobili* strain F4470
*Streptomyces albofaciens* strain AM16
*Streptomyces olivovhromogenes* strain IHB B13535
*Streptomyces avermitilis* strain CB2Z2
*Streptomyces gardneri* strain MML1736
*Streptomyces ochraceiscleroticus* strain R2

BLASTn results retrieved from the conventional PCR using *Streptomyces* - specific primers in gDNA extracted from adult scabies female mites and eggs.

**Table 2:** PCR primers used in the study.

Gene	Target organism	Primer name	Primer Sequence 5′-3′	Product size	Reference
wsp	*Wolbachia sp*.	Wol_Wsp_F	TGGTCCAATAAGTGATGAAGAAACTAGCTA	700 bp	^70^
		Wol_Wsp_R	AAAAATTAAACGCTACTCCAGCTTCTGCAC		
16S rRNA	*Wolbachia sp*.	Wol_16S_F	CGGGGGAAAAATTTATTGCT	589 bp	^71^
		Wol_16S_R	AGCTGTAATACAGAAAGTAAA		
16S rRNA	*Streptomyces sp*.	StrepB_F	ACAAGCCCTGGAAACGGGGT	519 bp	^72^
		StrepE_R	CACCAGGAATTCCGATCT		
		StrepF_R	ACGTGTGCAGCCCAAGACA	1074 bp	
16S rRNA (V1-V3)	Universal Eubacteria	27F	AGRGTTTGATCMTGGCTCAG	500 bp	^73^
		519R	GTNTTACNGCGGCKGCTG		
16S-23S rDNA (ITS)	*Klebsiella pneumoniae*	KP_PF	ATTTGAAGAGGTTGCAAACGAT	130 bp	^74^
		KP_PR	TTCACTCTGAAGTTTTCTTGTGTTC		

### Abundance of *Streptomyces* in adult female mites and eggs

**Figure 3:**
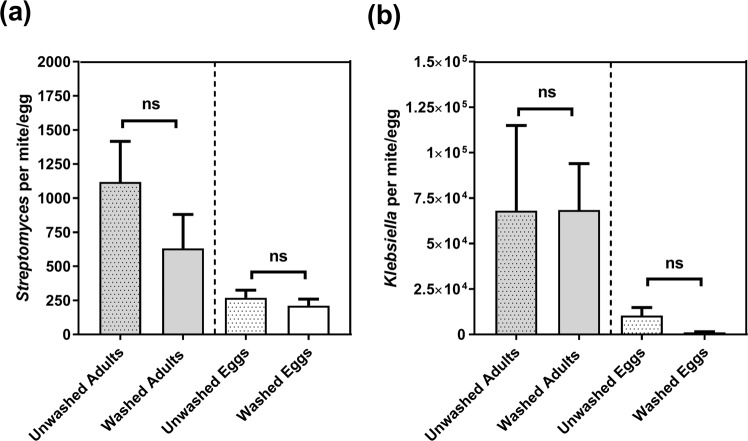
The number of (**a**) *Streptomyces sp*. and (**b**) *K*. *pneumoniae* present in washed vs. unwashed adult female scabies mites and eggs respectively. One ng of gDNA from 50–100 adult female mites or eggs was used as a template for duplex ddPCR using *Streptomyces* genus-specific and *K*. *pneumoniae* specific primers. Results are shown as means ± SEM from three independent experiments. The statistical significance of the differences between the numbers of bacteria in washed vs unwashed samples was estimated using 2 way ANOVA with Sidak’s multiple comparison test, ns: not significant.

In order to quantify the numbers of *Streptomyces sp*. compared to the highly abundant *K*. *pneumoniae* in adult female mites and in eggs, ddPCR was performed on genomic DNA separately prepared from 23 samples of mites and eggs respectively, with each sample containing approximately 100 mites or eggs. Washed vs. unwashed samples were included to obtain a preliminary estimate of the ratio of internal vs external bacteria. The numbers of bacteria were calculated based on the numbers of 16S rDNA copies per bacteria. Adult female mites contained more *Streptomyces sp*. and *K*. *pneumoniae* than eggs (Fig. 3a,b). In the DNA preparations from adult mites *K*. *pneumoniae* were present at a 10^4^ times higher abundance than *Streptomyces sp*. (Fig. 3a,b), whereas in egg preparations approximately 3 fold more *K*. *pneumoniae* than *Streptomyces sp*. were detected. There were no significant differences in the ratios of either bacteria between the washed and unwashed adults or between washed and unwashed eggs (Fig. 3a,b) indicating that, albeit much more abundance in adults, both bacteria could be present within and on the external surface of the mites and eggs. Nonetheless, the striking enrichment of *Streptomyces* relative to *K*. *pneumoniae* in the egg DNA preparations begged the question whether some of *Streptomyces* were likely to be within eggs.

### *Streptomyces sp*. and *K*. *pneumoniae* belong to the mite intestinal microflora and are detectable in the mite gut and in feces

**Figure 4:**
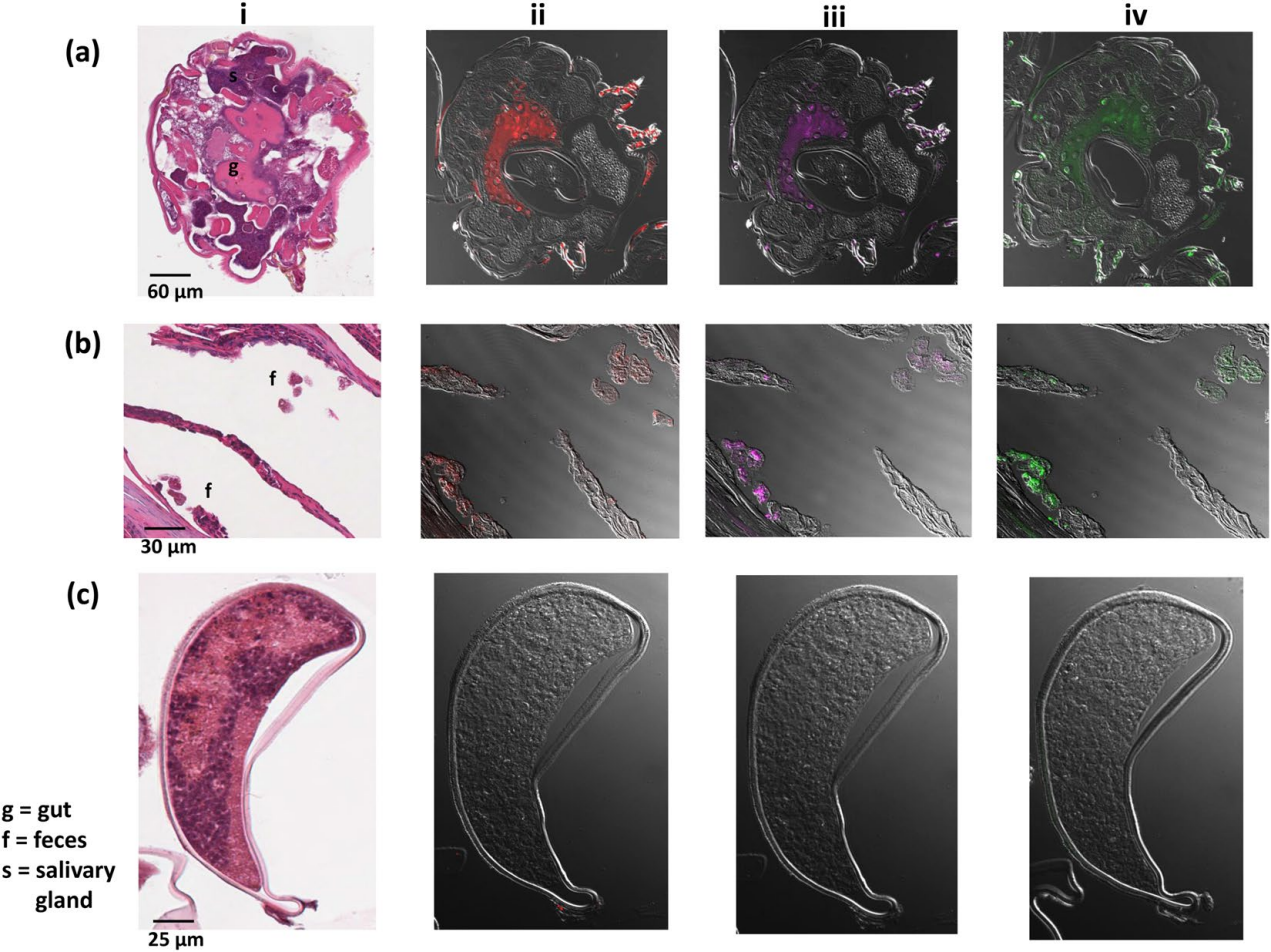
Visualization by Fluorescent *In Situ* hybridization (FISH) of *Streptomyces sp*. and *K*. *pneumoniae* within (**a**) an adult female scabies mite, (**b**) mite feces within epidermal burrows and (**c**) an egg. An adjacent section (i) was stained with Hematoxylin for orientation. The universal EUB338 probe (red, ii) was labelled with CalFlour590 while *Streptomyces sp*. (fuchsia, iii) and *K*. *pneumoniae* probes (green, iv) were both labelled with Cy5. Positive staining is visible in the gut area of the mite indicated as ‘g’ in section ‘i’ of panel ‘a’, and in the feces indicated as ‘f’ of panel (**b**).

To address the above question, Fluorescent *In Situ* hybridization (FISH) experiments were performed using probes against *Streptomyces* sp. and *K*. *pneumonia*. We found that these bacteria localized to the digestive tract and feces of the adult female scabies mites (Fig. 4a,b) and co-localized with a Eubacteria probe. None of these bacteria were detected within or on the surface of eggs (Fig. 4c), nor were they detected in the reproductive organs.

## Discussion

Studies investigating the function and influence of the microbiota on the biology and pathogenicity of the host organism have been reported for a number of free living mites and ticks, and for a few parasitic mites such as the sheep scab mite, *Psoroptes ovis*^35,36^ and the poultry mite *Dermanyssus gallinae*^24,37^. Here, we investigated the internal microbiome of scabies mites *S*. *scabiei* var. *suis*, as a model for the human biovar *S*. *scabiei* var. *hominis*. The literature differs in regards to the methods of removing external bacterial DNA from arthropods and how to analyse the microbiota. We therefore tested a range of reagents commonly used. We found that 0.65% Sodium hypochlorite solution was the most effective reagent at removing external bacteria. However, the metagenome data of the eggs still contained 0.09% bacterial sequences. This indicated that some bacteria were present within the egg, or not all external bacterial DNA was removed. Furthermore, samples with low microbial biomass, such as the egg samples in our current study, are especially prone to give skewed results owing to contaminations in reagents and sequencing kits^24,25^. In this study we could not unequivocally eliminate such exogenous contaminations. However, a detailed analysis of microbiome has revealed some interesting features as described below.

Among the most abundant genera in the egg dataset (Supplementary Table 2) were water- and soil- associated bacterial genera with *Bradyrhizobium*, *Sphinogoma*s, *Pseudomonas* and *Delftia* encompassing together almost half (48%) of the bacterial reads in the egg dataset. Some of the bacteria detected are considered as normal or pathogenic inhabitants of the skin or were previously reported as mite microbiota^6,38^. The remaining bacterial composition recovered from the DNA of washed eggs was overall consistent with the presence of Proteobacteria and Actinobacteria in the adult mites. The taxonomic profile of the egg bacterial metagenome may appear more diverse compared to the female metagenome, presumably because there is no overrepresented microbe taking up over 70% of the total bacterial read counts at the expense of other taxa.

The presence of *Klebsiella* and *Streptomyces* in the intestinal system of burrowing and feeding scabies mites is in agreement with findings from the other reported internal bacterial communities of synanthropic mites, for example *Dermatophagoides farina*, *Lepidoglyphus destructor* and *Tyrophagus putrescentiae*^39^. *Klebsiella* was the most abundant genus in the adult female scabies mite, with the majority belonging to the opportunistic pathogen *K*. *pneumoniae*. *Klebsiella sp*. are ubiquitous in nature and found in the environment and mucosal surfaces of pigs and humans^40^. *Klebsiella sp*. has been reported in the gut microbiota of Mediterranean fruit flies *Ceratitis capitate* and appeared to positively influence fecundity^41^. *K*. *pneumoniae* could play a similar role in the scabies mite biology, given its dominance in the microbiome and its residency in the mite gut. The clear co-localization of the *Streptomyces* and *Klebsiella* probes to the mite gut and feces of adult females, as well as, nymphs and larvae (data not shown), compared to their absolute absence from several hundred eggs individually examined under the confocal microscope, indicates that these bacteria are likely specifically adapted to the mite intestinal environment.

In alignment with a previous study^6^ *Corynebacteria* sequences were prominent in the adult female and egg microbiota data. Symbiotic *Corynebacteria* have been reported to form part of the gut microbiota of the alimentary systems of arthropods feeding on skin, such as *Triatoma infestans*^42^ and the tick species *Ixodes ricinus*, *Dermacentor reticulatus* and *Haemaphysalis concinna*^43^. Our study is also in agreement with the previous finding by Mounsey *et*. *al*. that a common endosymbiont of arthropods of the genus *Wolbachia* was absent in the scabies mite metagenome^44^.

An advantage of whole metagenome shotgun sequencing is the availability of species specific information about the organisms in a microbiota. Using this approach we could characterize the species of potential pathogenic genera including *Klebsiella*, *Streptococcus* and *Staphylococcus*. BLAST and Kraken analyses predicted *K*. *pneumonia* as the most abundant species in the female mite dataset. This bacterium is a common opportunistic pathogen isolated from blood^45^ and urine^46^ and is an important cause of multidrug resistance worldwide. We were able to identify a beta-lactamases gene fragment on reads assigned to *K*. *pneumoniae* (3 reads), indicating that the identified *K*. *pneumonia* in the female microbiome may carry resistance genes. Based on similarity searches we identified a read matching a plasmid, but no chromosomal sequence of *S*. *aureus*. Reads were similar (97% identity) to *Staphylococcus hyicus*, which is a porcine pathogen causing exudative epidermitis^47^, and to the common skin inhabitants *Staphylococcus sciuri*^48^, *Staphylococcus kloosii*^49^ and *Staphylococcus* *auricularis*^50^, which are associated with opportunistic infections^51^.

To the best of our knowledge *Streptomyces sp*. as a potential scabies mite symbiont has not been reported. *Streptomyces* are ubiquitous soil bacteria, and were only relatively recently recognized as symbiotic bacteria of plants and invertebrates^34^. *Streptomyces sp*. are known for their ability to produce antimicrobial compounds and enzymes that degrade complex carbohydrates such as cellulose^52–54^. In the context of *Streptomyces* as a possible symbiont of scabies mites and its localization to the intestinal system, it is tempting to speculate that these bacteria may be assisting the mite in digestion of skin and serum, which form a complex nutrient source. In addition, they may play a role in providing antimicrobial compounds to inhibit other bacteria unfavourable to the mite, thereby shaping the mite gut-specific microflora. For instance the presence of *Streptomyces* has been reported in the intestinal systems of various arthropods such as termites, beetles, millipedes, woodlice and earthworms^34^ and fascinating co-evolution has been proposed. *Streptomyces sp*. isolated from termite guts produce enzymes that degrade cellulose^53^ and lignin^54^ and thus provide the host with simplified nutrients. *Streptomyces* has been reported as a symbiont of European beewolves, where they reside in the specialized antennal glands and are applied to the brood cell prior to oviposition^55^. *Streptomyces* also produce multiple types of antimicrobial and antifungal compounds in order to protect beewolf larvae^56^. Similarly, fungus-growing ants (*Acromyrmex octospinosus*) are reported to harbour *Streptomyces* and the production of antimicrobial compounds is thought to protect the fungal garden from the attack of other parasitic bacteria and fungi^57^.

Vertical (transovarial) and horizontal transfer (via the environment) of symbiotic *Streptomyces* have been suggested^55,57,58^. From our FISH studies, we observed *Streptomyces* in the intestinal system of the burrowing stages, including adults, nymphs and larvae, and in excreted feces within the mite burrows, but not in the mite reproductive organs or eggs. A lack of fluorescence signal with the Eubacteria probe in eggs indicates that the internal tissue of the scabies eggs may be indeed sterile. Hence *Streptomyces* are unlikely to be transmitted from the mother to progeny via the transovarial route. We propose that hatched larvae take up *Streptomyces* from their microenvironment when they feed within the epidermal burrows.

Despite the striking similarities with respect to general structure, physiology and immunology between the skin of pigs and humans, their respective skin microbiota will be unique. While clinical manifestation of *S*. *scabiei* infection of pig and human are highly comparable (hence pigs are the chosen scabies animal model) the scabies associated host skin microbiota of pigs and human will be found to be different. Symbiotic bacteria, essential for parasite survival, likely have no direct influence on the host skin microbiota and scabies-associated skin pathology. However, understanding and targeting the interactions between mites and their symbionts could be a novel avenue to develop control measures for scabies infection. Following this agenda the next step would be to address the specific roles of *Streptomyces* and other mite-specific microbiota and to experimentally remove candidate symbiotic bacteria by specific antibiotic treatments. Such experiments should reveal their influence on mite survival and may indicate what niche they occupy within the parasite.

## Methods

### Ethics statement

Animal care and handling procedures used in this study followed the Animal Care and Protection Act, in compliance with the Australian code of practice for the care and use of animals for scientific purposes, outlined by the Australian National Health and Medical Research Council. The study was approved by the Animal Ethics Committees of the QIMR Berghofer Medical Research Institute (P630, QIMRB A0306-621M) and the Queensland Animal Science Precinct (QASP), University of Queensland, Gatton Campus (DAFF-AEC SA 2015/03/504).

### Collection of mites and eggs

Skin crust samples containing *S*. *scabiei* var. *suis* were obtained from our porcine scabies model (*Sus scrofa domesticus*) housed and maintained at QASP, UQ Gatton Campus. Crusts were divided into glass petri dishes, which were humidified with a moist paper pad, sealed with parafilm, covered with an aluminium foil and warmed overnight on a base light of a dissecting microscope at RT. This encourages the mites to crawl out of the crusts. For the preparation of genomic DNA for metagenome sequencing, groups of 2000 adult female mites and 4000 eggs were collected under a dissection microscope into sterile 2 ml reinforced microfuge tubes (MK28, Precellys®). For preparation of gDNA for PCR, groups of 50–100 adult female mites or eggs were collected into sterile 1.5 ml microfuge tubes (BioMasher II®).

### Establishing the washing technique to remove external DNA from mites and eggs

Four different cleaning solutions were tested: (1) 0.65% Sodium hypochlorite solution, (2) 4% Paraformaldehyde solution, (3) 70% Ethanol solution (V/V) and (4) DNase/Lysozyme mixture (2U RNase-free DNase, Promega) and 0.6 µg/ml lysozyme (Sigma-Aldrich) Promega RQ1 RNase-Free DNase Reaction Buffer respectively. For washes in solutions (1) to (3) 5 groups of 10 eggs each (n = 5) were collected into 0.2 ml microfuge tubes containing cleaning solution and agitated at 750 rpm for 7 min at RT. Samples were centrifuged at 30,000 × *g* for 3 min and the supernatant was removed. This washing process was repeated 2 times and eggs were rinsed twice with 200 µl of MQ water by agitation at 750 rpm for 7 min at RT followed by centrifugation at 30,000 × *g* for 3 min and supernatant was removed.

For samples treated with DNase/Lysozyme mixture (4), 10 eggs were added to 50 µl of DNase/Lysozyme solution and incubated at 37 °C for 30 min at 200 rpm (Bioline Orbital incubator shaker). Eggs were centrifuged at 30,000 × *g* for 3 min and supernatant was removed. Eggs were washed with 200 µl of MQ water by agitation at 750 rpm for 7 min at RT followed by centrifugation at 30,000 × *g* for 3 min. After addition of 200 µl of MQ water and agitation at 750 rpm for 7 min at RT, eggs were incubated at 65 °C for 10 min to inactivate the DNase. Samples were then centrifuged at 30,000 × *g* for 3 min and supernatant was removed.

### Homogenization of mites and eggs

Mites and eggs were homogenized immediately prior to DNA extraction. Eggs were homogenized with a cordless hand-held homogenizer (Pellet Pestle®, Kontes) using sterile pellet pestles (Kimble Chase). Mites were homogenized by bead-beating using 6 lots of 2.8 mm stainless steel beads (Precellys®) in a tube with 1 ml ice cold G2 buffer (Qiagen) in a Precellys®24 tissue homogenizer (Bertin Technologies) at 6,800 rpm for 3 × 30 s cycles with 30 s rests between each cycle at 4 °C. Tubes were centrifuged at 10, 000 × *g* to reduce foam and stainless steel beads were removed with sterile tweezers.

### DNA extraction and precipitation

DNA from large pools of washed adult female mites or eggs was extracted using the Qiagen Blood and Tissue Mini kit (Qiagen) according to the manufacturer’s instructions with minor modifications. 800 μl of ice cold G2 buffer and 100 µl of Proteinase K were added to the tubes. RNaseA was added to the final concentration of 0.2 mg/ml and samples were incubated in a water bath for 1.5 h at 56 °C. Samples were centrifuged at 4000 × *g* for 10 min at 4 °C. Five hundred µl of lysate was transferred into a Min-Elute spin column (Qiagen), centrifuged at 6,000 × *g* for 1 min, and the flow through was discarded. This process was repeated with the remaining cell lysate. The column was washed with 500 µl buffer AW1 by centrifugation at 6,000 × *g* for 1 min and the flow through was discarded. The column was washed again with 500 µl AW2 by centrifugation at 18,000 × *g* for 3 min and the flow through was discarded. DNA was eluted into a clean Eppendorf tube with 25 µl buffer AE, incubated at RT for 2 min, followed by centrifugation at 18,000 × *g* for 2 min. To achieve high DNA concentration required for metagenome sequencing, extracted DNA was pooled and precipitated by adding 0.1% Sodium acetate (3M, pH 5.2), 1 µl of glycogen and 2 volumes of 100% EtOH and storing overnight at −80 °C. Samples were centrifuged for 30 min at 18,000 × *g* at 4 °C, supernatant was removed, and the pellet was washed in 1 ml of 70% EtOH on ice for 5 min. Samples were centrifuged at 18,000 × *g* at 4 °C for 15 min, the supernatant was removed and the pellet air-dried and re-suspended in sterile TE buffer. For extraction of DNA from small pools (50–100 mites/eggs) samples, the same protocol for DNA homogenization and extraction was followed. Concentration of DNA was measured using the Quant-iT™ PicoGreen® dsDNA Reagent and Kits (Invitrogen) according to the manufacturer’s instructions.

### Analysis of various washing techniques by qPCR

Quantification of 16S rDNA was performed by qPCR in a Roche LightCycler® 480 with universal eubacteria primers (Table 2) to detect all bacteria present in samples of 5 eggs. Five samples per treatment (n = 5) were subjected to qPCR in duplicate, and a positive control (1.54 × 10^7^ copies/reaction), a no-template control and extraction controls were included. A dilution standard ranging from 1.54 × 10^8^ to 1.54 × 10^0^ copies/reaction was also included, to create a standard curve for absolute quantification. On completion of the qPCR, melting curve analyses and 2^nd^ derivative maximum analyses for absolute quantification were performed as outlined in the LightCycler® 480 Instrument Operator’s Manual, LightCycler® 480 Software, Version 1.5 (Roche). A single internal standard was used to fit the externally generated standard curve prior to calculating the absolute copy number and Crossing point (Cp) values for each sample. The statistical significance was calculated by 1-way ANOVA, Dunnett’s multiple comparisons test. PCR products were analyzed by gel electrophoresis.

### Metagenome sequencing

The metagenomes of washed scabies mites and eggs were sequenced by Mr. DNA Molecular Research (Shallowater, TX, USA). Libraries were prepared using Nextera® DNA Sample Preparation Kit (Illumina, San Diego, CA, USA). Fifty ng of gDNA (2.4 ng/ul) was fragmented and end-tagged with an adaptor sequence. Tagged fragments were purified and amplified in a limited cycle PCR (5 cycles). The Qubit® dsDNS HS Assay Kit (Molecular Probes, Life Technologies) was used to measure the library concentration and the average library size was determined by Agilent 2100 Bioanalyzer (Agilent Technologies, Santa Clara, CA, USA). Libraries were pooled in equimolar ratios of 2 nM and 11 pM of library was clustered using the cBot (Illumina) and sequenced paired end for 300 cycles by HiSeq2500 system (Illumina).

### Data processing and analysis

To generate pure scabies draft genomes without bacteria sequences, the published scabies draft genomes of *S*. *scabiei* (var. *suis*, var. *hominis* and var. *canis*)^59,60^ were re-analyzed. Putative chimeric scaffolds combining sequences from scabies and bacteria were excluded if at least 25% of the scaffold sequence matched a bacterial reference with at least 90% nucleotide identity using BLAST (NCBI bacterial genome database September 2016)^61^. Illumina paired-end reads derived from the mite and egg preparations generated here were quality trimmed using the software Trimmomatic^62^ with a quality threshold of 20 and minimum read length of 30 nucleotide base pairs. Scabies mite sequences were filtered out by mapping these metagenome reads to the re-analyzed scabies draft genomes. Porcine host sequences were excluded by screening the pig draft genome (*S*. *scrofa*, draft assembly version Sscrofa10.2)^63^ using the Burrows-Wheeler Aligner’s Smith-Waterman Alignment algorithm (BWA-SW)^64^. The remaining unmapped reads were classified with the software Kraken v2.0.6^65^ using the paired-end mode. The taxonomic composition was visualized using the software Krona plots^66^. Metagenome reads matching the beta-lactamase OKB gene (AM051161.1) were identified using BLAST. Reads matching *Streptococcus* or *Staphylococcus* taxa using the software Kraken were analyzed for the best species matches against the NCBI nucleotide database using BLAST.

### Conventional and digital droplet PCR (ddPCR)

Conventional PCR was performed using AmpliTag Gold^®^ DNA polymerase kit (Life Technologies^TM^) according to the manufacturer’s instructions to detect *Streptomyces sp*. and *Wolbachia sp*. using various sets of primers (Table 1). PCR detection for *Klebsiella pneumoniae* was included as a positive control, due to the high abundance of this bacterium in the metagenome of adult female mites. gDNA of mosquito infected with *Wolbachia* (*Aedes aegypti* strain wMel2)^33^, *Streptomyces avermitilis* ATCC3237 (provided by Professor Rob Capon, University of Queensland) and *K*. *pneumoni*ae 8cb53 (provided by Pathology Laboratory, Queensland Health) were used as positive controls and for optimization of PCR conditions. PCR was performed on the gDNAs extracted from 5 samples of thousands of mites of various developmental stages including eggs and 10 samples of 50–100 mites or 50–100 eggs. PCR conditions were slightly varied for the detection of each type of bacteria. For *Streptomyces sp*., PCR reactions were denatured for 10 min at 94 °C, followed by 35 cycles each of 94 °C for 45 s, 50 °C for 40 s and 72 °C for 2 min, followed by an extension period at 72 °C for 10 min. For *Wolbachia sp*., PCR reactions were denatured for 10 min at 94 °C, followed by 35 cycles each of 94 °C for 10 s, 54 °C for 30 s and 72 °C for 45 s, followed by an extension period at 72 °C for 10 min. For *K*. *pneumoniae*, PCR reactions were denatured for 10 min at 94 °C, followed by 35 cycles each of 94 °C for 30 s, 57 °C for 20 s and 72 °C for 20 s, followed by an extension period at 72 °C for 10 min.

Duplex ddPCR was performed to quantify *Streptomyces sp*. and *K*. *pneumoniae* using ddPCR^TM^ supermix for probes without dUTP according to the manufacturer’s instructions (BIO-RAD). One ng of scabies mite or egg gDNA was used as a template. PCR reactions were denatured for 5 min at 95 °C, followed by 35 cycles each of 95 °C for 30 s, 58 °C for 1 min and 72 °C for 1 min, followed by one cycle each at 72 °C for 10 min, 4 °C for 5 min and 90 °C for 5 min. Primers used are listed in Table 2.

### Fluorescent *In Situ* hybridization (FISH)

Batches of between 50 and 200 scabies mites or eggs were collected from porcine scabetic skin. Isolated mites and eggs and also whole crust pieces containing parasites were fixed by soaking in 4% paraformaldehyde solution for 1 h at RT and embedded in paraffin blocks. Four µm sections from these blocks were cut under aseptic condition and placed on sterile DNase/RNase free Uberfrost Plus glass slides (InstrumeC). Slides were deparaffinized in RNase free solutions of 100% Xylene for 2 × 10 min, 100% Ethanol for 2 × 5 min, 95% Ethanol for 2 × 5 min, 70% Ethanol for 5 min, then rinsed 3 x in MQ water. Samples were treated with proteinase K (20 µg/ml, 0.05 M TBS, 0.01 M calcium chloride, pH 8.0) at RT for 10 min, then rinsed 3 x with MQ water. Slides were dehydrated in 70% Ethanol for 2 × 5 min, 95% Ethanol for 2 × 5 min and air dried. Slides containing two adjacent sections were hybridized with a mixture of two probes each at 100 nM concentration for 16–20 h at 45 °C in the dark. Probes were labelled at the 5′ end with either CalFlour590, which excites at 566 nm and emits at 588 nm or Cy5, which excites at 633 nm and emits at 670 nm. The combinations used were EUB338 (5′CalFluor590-GCTGCCTCCCGTAGGAGT-3′)^67^ +STREPM (5′Cy5- CCGGGTCTGCATTCGATACGGGCAGACT-3′) to detect Eubacteria and *Streptomyces sp*., or EUB338 + Kpn23S (5′ Cy5- CCT ACA CAC CAG CGT GCC -3′)^68^ to detect Eubacteria and *K*. *pneumoniae*. Slides were washed with SSC buffer (Sigma) containing 10 mM DTT for 3 × 15 min at 55 °C, 1 × 10 min at RT with 0.2 µg/ml DAPI (Sigma) to stain nuclei and 1 × 10 min at RT. Slides were rinsed twice in MQ water and a coverslip was mounted with glycerol gelatine mounting medium (Sigma). Mites and associated bacteria were visualized under a Zeiss 780 NLO scanning confocal microscope. To assist orientation, sections adjacent to the sections analyzed by FISH were Haematoxylin stained^69^.

## Supplementary information

Supplementary Dataset 1

Supplementary Dataset 2

## Data Availability

The raw Illumina sequencing data generated in this study are available from NCBI SRA, bio project number PRJNA513944 with BioSamples accessions SAMN10720096 (eggs) and SAMN10720097 (adult females).

## References

[1] Fischer K, Holt D, Currie B, Kemp D (2012). Scabies: important clinical consequences explained by new molecular studies. Adv. Parasitol..

[2] Reynolds SL (2014). Scabies mite inactive serine proteases are potent inhibitors of the human complement lectin pathway. PLoS. Negl. Trop. Dis..

[3] Mika A (2012). Novel scabies mite serpins inhibit the three pathways of the human complement system. PLoS One.

[4] Swe PM, Fischer K (2014). A scabies mite serpin interferes with complement-mediated neutrophil functions and promotes staphylococcal growth. PLoS. Negl. Trop. Dis..

[5] Swe PM, Christian LD, Lu HC, Sriprakash KS, Fischer K (2017). Complement inhibition by Sarcoptes scabiei protects Streptococcus pyogenes - An *in vitro* study to unravel the molecular mechanisms behind the poorly understood predilection of S. pyogenes to infect mite-induced skin lesions. PLoS. Negl. Trop. Dis..

[6] Swe PM, Zakrzewski M, Kelly A, Krause L, Fischer K (2014). Scabies mites alter the skin microbiome and promote growth of opportunistic pathogens in a porcine model. PLoS Negl Trop Dis.

[7] Swe PM, Reynolds SL, Fischer K (2014). Parasitic scabies mites and associated bacteria joining forces against host complement defence. Parasite Immunol..

[8] Hay RJ (2003). Pyoderma and scabies: a benign association?. Curr. Opin. Infect. Dis..

[9] Engelman D (2013). Toward the global control of human scabies: introducing the International Alliance for the Control of Scabies. PLoS. Negl. Trop. Dis..

[10] Lynar S, Currie BJ, Baird R (2017). Scabies and mortality. Lancet Infect. Dis..

[11] Bowen AC, Tong SY, Chatfield MD, Carapetis JR (2014). The microbiology of impetigo in indigenous children: associations between *Streptococcus pyogenes*, *Staphylococcus aureus*, scabies, and nasal carriage. BMC Infect. Dis..

[12] Thornley S (2018). Scabies is strongly associated with acute rheumatic fever in a cohort study of Auckland children. J. Paediatr. Child Health.

[13] Romani L (2015). Scabies and impetigo prevalence and risk factors in Fiji: a national survey. PLoS. Negl. Trop. Dis..

[14] Carapetis, J., Steer, A. C. & Mulholland, E. K. The current evidence for the burden of group A streptococcal diseases. *World Health Organization*, *Discussion papers of child health* (2005).

[15] Carapetis JR, Connors C, Yarmirr D, Krause V, Currie BJ (1997). Success of a scabies control program in an Australian aboriginal community. Pediatr. Infect. Dis. J.

[16] Whitehall J, Kuzulugil D, Sheldrick K, Wood A (2013). Burden of paediatric pyoderma and scabies in North West Queensland. J. Paediatr. Child Health.

[17] Clucas, D. B. *et al*. Disease burden and health-care clinic attendances for young children in remote aboriginal communities of northern Australia. *Bull*. *World Health Organ*. **86**, 275–281, doi:S0042-96862008000400012 [pii] (2008). 10.2471/BLT.07.043034 PMC2647416 18438516

[18] Lawrence, G. *et al*. Control of scabies, skin sores and haematuria in children in the Solomon Islands: another role for ivermectin. *Bull*. *World Health Organ*. **83**, 34–42, doi:S0042-96862005000100012 (2005). PMC2623469 15682247

[19] Steere AC, Broderick TF, Malawista SE (1978). Erythema chronicum migrans and Lyme arthritis: epidemiologic evidence for a tick vector. Am. J. Epidemiol..

[20] Evans ME (1985). Francisella tularensis. Infect Control.

[21] Shelley WB, Shelley ED, Burmeister V (1988). *Staphylococcus aureus* colonization of burrows in erythrodermic Norwegian scabies. A case study of iatrogenic contagion. J. Am. Acad. Dermatol..

[22] Su Q (2014). The endosymbiont Hamiltonella increases the growth rate of its host *Bemisia tabaci* during periods of nutritional stress. PLoS One.

[23] Egert M, Wagner B, Lemke T, Brune A, Friedrich MW (2003). Microbial community structure in midgut and hindgut of the humus-feeding larva of *Pachnoda ephippiata* (Coleoptera: Scarabaeidae). Appl. Environ. Microbiol..

[24] Shinzato N, Muramatsu M, Matsui T, Watanabe Y (2007). Phylogenetic analysis of the gut bacterial microflora of the fungus-growing termite *Odontotermes formosanus*. Biosci. Biotechnol. Biochem..

[25] Senderovich Y, Halpern M (2013). The protective role of endogenous bacterial communities in chironomid egg masses and larvae. ISME J..

[26] Koga R, Tsuchida T, Sakurai M, Fukatsu T (2007). Selective elimination of aphid endosymbionts: effects of antibiotic dose and host genotype, and fitness consequences. FEMS Microbiol. Ecol..

[27] Hardie J, Leckstein P (2007). Antibiotics, primary symbionts and wing polyphenism in three aphid species. Insect Biochem. Mol. Biol..

[28] Bandi C (1999). Effects of tetracycline on the filarial worms Brugia pahangi and *Dirofilaria immitis* and their bacterial endosymbionts. Wolbachia. Int. J. Parasitol..

[29] Saldana MA, Hegde S, Hughes GL (2017). Microbial control of arthropod-borne disease. Mem. Inst. Oswaldo Cruz.

[30] Mounsey K (2010). A Tractable Experimental Model for Study of Human and Animal Scabies. PLoS. Negl. Trop. Dis..

[31] Fain A (1978). Epidemiological problems of scabies. Int. J. Dermatol..

[32] Arlian LG, Morgan MS (2017). A review of *Sarcoptes scabiei*: past, present and future. Parasites & vectors.

[33] Mofiz E (2016). Mitochondrial Genome Sequence of the Scabies Mite Provides Insight into the Genetic Diversity of Individual Scabies Infections. PLoS. Negl. Trop. Dis..

[34] Seipke RF, Kaltenpoth M, Hutchings MI (2012). *Streptomyces* as symbionts: an emerging and widespread theme?. FEMS Microbiol. Rev..

[35] Hogg JC, Lehane MJ (1999). Identification of bacterial species associated with the sheep scab mite (*Psoroptes ovis*) by using amplified genes coding for 16S rRNA. Appl. Environ. Microbiol..

[36] Hogg JC, Lehane MJ (2001). Microfloral diversity of cultured and wild strains of *Psoroptes ovis* infesting sheep. Parasitology.

[37] Valiente Moro C (2009). The poultry red mite (*Dermanyssus gallinae*): a potential vector of pathogenic agents. Exp. Appl. Acarol..

[38] Chan TF (2015). The draft genome, transcriptome, and microbiome of *Dermatophagoides farinae* reveal a broad spectrum of dust mite allergens. J. Allergy. Clin. Immunol..

[39] Hubert J (2012). Detection and identification of species-specific bacteria associated with synanthropic mites. Microb. Ecol..

[40] Podschun R, Ullmann U (1998). *Klebsiella* spp. as nosocomial pathogens: epidemiology, taxonomy, typing methods, and pathogenicity factors. Clin. Microbiol. Rev..

[41] Ben Ami E, Yuval B, Jurkevitch E (2010). Manipulation of the microbiota of mass-reared Mediterranean fruit flies *Ceratitis capitata* (Diptera: Tephritidae) improves sterile male sexual performance. ISME J..

[42] Gumiel M (2015). Characterization of the microbiota in the guts of *Triatoma brasiliensis* and *Triatoma pseudomaculata* infected by *Trypanosoma cruzi* in natural conditions using culture independent methods. Parasit. Vectors.

[43] Rudolf I (2009). 16S rRNA gene-based identification of cultured bacterial flora from host-seeking *Ixodes ricinus*, *Dermacentor reticulatus* and *Haemaphysalis concinna* ticks, vectors of vertebrate pathogens. Folia. Microbiol. (Praha).

[44] Mounsey KE (2005). Analysis of *Sarcoptes scabiei* finds no evidence of infection with Wolbachia. Int. J. Parasitol..

[45] Brisse S, Passet V, Grimont PA (2014). Description of *Klebsiella quasipneumoniae* sp. nov., isolated from human infections, with two subspecies, *Klebsiella quasipneumoniae subsp*. *quasipneumoniae subsp*. *nov*. *and Klebsiella quasipneumoniae subsp*. *similipneumoniae subsp*. *nov*., *and demonstration that Klebsiella singaporensis is a junior heterotypic synonym of Klebsiella variicola*. Int. J. Syst. Evol. Microbiol..

[46] Elliott, A. G., Ganesamoorthy, D., Coin, L., Cooper, M. A. & Cao, M. D. Complete Genome Sequence of *Klebsiella quasipneumoniae* subsp. *similipneumoniae* Strain ATCC 700603. *Genome Announc*. **4**, 10.1128/genomeA.00438-16 (2016). 10.1128/genomeA.00438-16 PMC4882950 27231369

[47] Park J, Friendship RM, Poljak Z, Weese JS, Dewey CE (2013). An investigation of exudative epidermitis (greasy pig disease) and antimicrobial resistance patterns of *Staphylococcus hyicus* and *Staphylococcus aureus* isolated from clinical cases. Can. Vet. J..

[48] Stepanovic S (2005). Identification and characterization of clinical isolates of members of the *Staphylococcus sciuri* group. J. Clin. Microbiol..

[49] Schleifer KH, Kilpper-Bälz R, Devriese LA (1984). *Staphylococcus arlettae* sp. nov., *S*. *equorum* sp. nov. and S. *kloosii* sp. nov. Three New Coagulase-Negative, Novobiocin-Resistant Species from Animals. Syst. Appl. Microbiol..

[50] Kloos WE, Schleifer KH (1983). *Staphylococcus auricularis* sp. nov.: an Inhabitant of the Human External Ear†. Int. J. Syst. Evol. Microbiol..

[51] Chen S (2007). A highly pathogenic strain of *Staphylococcus sciuri* caused fatal exudative epidermitis in piglets. PLoS One.

[52] Arango RA (2016). Antimicrobial Activity of Actinobacteria Isolated From the Guts of Subterranean Termites. Environ. Entomol..

[53] Schafer A (1996). Hemicellulose-degrading bacteria and yeasts from the termite gut. J. Appl. Bacteriol..

[54] Pasti MB, Pometto AL, Nuti MP, Crawford DL (1990). Lignin-solubilizing ability of actinomycetes isolated from termite (Termitidae) gut. Appl. Environ. Microbiol..

[55] Kaltenpoth M, Gottler W, Herzner G, Strohm E (2005). Symbiotic bacteria protect wasp larvae from fungal infestation. Curr. Biol..

[56] Kroiss J (2010). Symbiotic *Streptomycetes* provide antibiotic combination prophylaxis for wasp offspring. Nat. Chem. Biol..

[57] Barke J (2010). A mixed community of actinomycetes produce multiple antibiotics for the fungus farming ant *Acromyrmex octospinosus*. BMC Biol..

[58] Kaltenpoth M, Yildirim E, Gurbuz MF, Herzner G, Strohm E (2012). Refining the roots of the beewolf-*Streptomyces* symbiosis: antennal symbionts in the rare genus *Philanthinus* (Hymenoptera, Crabronidae). Appl. Environ. Microbiol..

[59] Mofiz E (2016). Genomic resources and draft assemblies of the human and porcine varieties of scabies mites, *Sarcoptes scabiei* var. *hominis* and var. *suis*. Gigascience.

[60] Rider SD, Morgan MS, Arlian LG (2015). Draft genome of the scabies mite. Parasit. Vectors.

[61] Altschul SF (1997). Gapped BLAST and PSI-BLAST: a new generation of protein database search programs. Nucleic Acids Res..

[62] Bolger AM, Lohse M, Usadel B (2014). Trimmomatic: a flexible trimmer for Illumina sequence data. Bioinformatics.

[63] Groenen MA (2012). Analyses of pig genomes provide insight into porcine demography and evolution. Nature.

[64] Li H, Durbin R (2010). Fast and accurate long-read alignment with Burrows-Wheeler transform. Bioinformatics.

[65] Wood DE, Salzberg SL (2014). Kraken: ultrafast metagenomic sequence classification using exact alignments. Genome Biol..

[66] Ondov BD, Bergman NH, Phillippy AM (2011). Interactive metagenomic visualization in a Web browser. BMC Bioinformatics.

[67] Amann RI (1990). Combination of 16S rRNA-targeted oligonucleotide probes with flow cytometry for analyzing mixed microbial populations. Appl. Environ. Microbiol..

[68] Alm EW, Oerther DB, Larsen N, Stahl DA, Raskin L (1996). The oligonucleotide probe database. Appl. Environ. Microbiol..

[69] Lillie, R. D. *Histopathologic technic and practical histochemistry*. 3rd ed. edn, (Blakiston Division, McGraw-Hill, 1975).

[70] Jeyaprakash A, Hoy MA (2000). Long PCR improves *Wolbachia* DNA amplification: wsp sequences found in 76% of sixty-three arthropod species. Insect. Mol. Biol..

[71] Heddi, A., Grenier, A. M., Khatchadourian, C., Charles, H. & Nardon, P. Four intracellular genomes direct weevil biology: nuclear, mitochondrial, principal endosymbiont, and Wolbachia. *Proc. Natl. Acad. Sci. USA* **96**, 6814–6819 (1999). 10.1073/pnas.96.12.6814 PMC21998 10359795

[72] Rintala H, Nevalainen A, Ronka E, Suutari M (2001). PCR primers targeting the 16S rRNA gene for the specific detection of streptomycetes. Mol Cell Probes.

[73] Dowd SE (2008). Evaluation of the bacterial diversity in the feces of cattle using 16S rDNA bacterial tag-encoded FLX amplicon pyrosequencing (bTEFAP). BMC Microbiol..

[74] Liu Y (2008). PCR detection of *Klebsiella pneumoniae* in infant formula based on 16S-23S internal transcribed spacer. Int. J. Food Microbiol..

