# Supplementary material for: High-throughput metagenome analysis of the Sarcoptes scabiei internal microbiota and in-situ identification of intestinal Streptomyces sp
Source: Sci Rep. 2019 Aug 13;9:11744. doi: 10.1038/s41598-019-47892-0 (PMC6692375; doi:10.1038/s41598-019-47892-0)
Supplement: Supplementary file 1 — Supplementary Dataset 1 [file 41598_2019_47892_MOESM1_ESM.docx]

**High-throughput metagenome analysis of the *Sarcoptes scabiei* internal microbiota and *in-situ* identification of intestinal *Streptomyces sp*.**

**^1^Pearl M. Swe^+^, ^2^Martha Zakrzewski^+^, ^1^Rebecca Waddell, ^1^Kadaba S. Sriprakash and ^1^Katja Fischer**

**S1 Table 1. Top genera in the female adult microbiome predicted from the PE reads using Kraken**

| Read counts | Genus (Phylum) | Reagent contami-nation | Soil or aquatic habitats | Pathogen in mammals | Symbiont in arthropods |
| --- | --- | --- | --- | --- | --- |
| 18748 | Klebsiella (phylum Proteobacteria) |  | ^1^ | ^2^ |  |
| 2087 | Corynebacterium (phylum Actinobacteria) | ^3^ |  |  | ^4^ |
| 594 | Escherichia (phylum Proteobacteria) | ^3^ |  | ^5^ |  |
| 78 | Bradyrhizobium (phylum Proteobacteria) | ^3^ |  |  |  |
| 61 | Staphylococcus (phylum Firmicutes) | ^3^ |  |  |  |
| 61 | Lacinutrix (phylum Bacteroidetes) |  | ^6^ |  |  |
| 36 | Pseudoalteromonas (phylum Proteobacteria) |  | ^7^ |  |  |
| 29 | Sphingomonas (phylum Proteobacteria) | ^3^ |  |  |  |
| 29 | Arenibacter (phylum Bacteroidetes) |  | ^8^ |  |  |
| 28 | Colwellia (phylum Proteobacteria) |  | ^9,10^ |  |  |
| 24 | Pseudomonas (phylum Proteobacteria) | ^3^ |  |  |  |
| 23 | Acidovorax (phylum Proteobacteria) | ^3^ |  |  |  |
| 17 | Citrobacter (phylum Proteobacteria)^1^ |  | ^11^ |  |  |
| 16 | Enterobacter (phylum Proteobacteria) | ^3^ | ^12^ |  |  |
| 15 | Polaribacter (phylum Bacteroidetes) |  | ^13^ |  |  |
| 13 | Flavobacterium (phylum Bacteroidetes) | ^3^ |  |  |  |
| 13 | Dietzia (phylum Actinobacteria) | ^3^ |  |  |  |
| 12 | Salmonella (phylum Proteobacteria) |  | ^14^ | ^15^ |  |
| 10 | Delftia (phylum Proteobacteria) | ^3^ |  |  |  |
| 9 | Streptomyces (phylum Actinobacteria) |  | ^16^ |  | ^17^ |
| 9 | Marinomonas (phylum Proteobacteria) |  | ^18^ |  |  |
| 9 | Haliscomenobacter (phylum Bacteroidetes) |  | ^19^ |  |  |
| 6 | Oleispira (phylum Proteobacteria) |  | ^20^ |  |  |
| 6 | Kocuria (phylum Actinobacteria) | ^3^ |  |  |  |
| 6 | Cellulophaga (phylum Bacteroidetes) |  | ^21^ |  |  |
| 5 | Vibrio (phylum Proteobacteria) |  | ^22^ | ^23^ |  |
| 5 | Tenacibaculum (phylum Bacteroidetes) |  | ^24^ |  |  |
| 5 | Tamlana (phylum Bacteroidetes) |  | ^25^ |  |  |
| 5 | Siansivirga (phylum Bacteroidetes) |  | ^26^ |  |  |
| 5 | Burkholderia (phylum Proteobacteria) | ^3^ |  | ^27^ |  |

**S1 Table 2. Top genera in the egg microbiome predicted from the PE reads using Kraken.**

| Read counts | Genus (phylum) | Reagent contami-nation | Soil or aquatic habitats | Pathogen in mammals | Symbiont in arthropods |
| --- | --- | --- | --- | --- | --- |
| 662 | Sphingomonas (phylum Proteobacteria) | ^3^ |  |  |  |
| 653 | Bradyrhizobium (phylum Proteobacteria) | ^3,28,29^ |  |  |  |
| 254 | Corynebacterium (phylum Actinobacteria) | ^3,28^ |  |  | ^4^ |
| 89 | Pseudomonas (phylum Proteobacteria) | ^3,28^ |  |  |  |
| 68 | Lacinutrix (phylum Bacteroidetes) |  | ^30^ |  |  |
| 59 | Delftia (phylum Proteobacteria) | ^3^ |  |  |  |
| 41 | Klebsiella (phylum Proteobacteria) | ^28^ | ^1^ | ^2^ |  |
| 28 | Arenibacter (phylum Bacteroidetes) |  | ^31^ |  |  |
| 27 | Acidovorax (phylum Proteobacteria) | ^3^ |  |  |  |
| 25 | Sphingobium (phylum Proteobacteria) | ^3^ |  |  |  |
| 24 | Burkholderia (phylum Proteobacteria) | ^3,28^ |  | ^27^ |  |
| 23 | Streptomyces (phylum Actinobacteria) |  | ^16^ |  | ^17^ |
| 22 | Pseudoalteromonas (phylum Proteobacteria) |  | ^7^ |  |  |
| 21 | Stenotrophomonas (phylum Proteobacteria) | ^3,28^ |  |  |  |
| 19 | Colwellia (phylum Proteobacteria) |  | ^9,10^ |  |  |
| 16 | Lysobacter (phylum Proteobacteria) |  | ^32^ |  |  |
| 15 | Microbacterium (phylum Actinobacteria) | ^3^ |  |  |  |
| 15 | Methylobacterium (phylum Proteobacteria) | ^3,28^ |  |  |  |
| 14 | Cutibacterium (phylum Actinobacteria) | ^3^ |  |  |  |
| 13 | Streptococcus (phylum Firmicutes) | ^3,28^ |  | ^33^ |  |
| 13 | Staphylococcus (phylum Firmicutes) | ^28^ |  | ^34,35^ |  |
| 13 | Massilia (phylum Proteobacteria) | ^3,28^ |  |  |  |
| 13 | Dyella (phylum Proteobacteria) | ^36^ |  |  |  |
| 13 | Azospira (phylum Proteobacteria) | ^3^ |  |  |  |
| 12 | Xanthomonas (phylum Proteobacteria) | ^3^ |  |  |  |
| 12 | Polaribacter (phylum Bacteroidetes) |  | ^13^ |  |  |
| 12 | Flavobacterium (phylum Bacteroidetes) | ^3^ |  |  |  |
| 10 | Variovorax (phylum Proteobacteria) | ^3^ |  |  |  |
| 10 | Sphingopyxis (phylum Proteobacteria) | ^3^ |  |  |  |
| 10 | Rhodopseudomonas (phylum Proteobacteria) |  | ^37^ |  |  |
| 10 | Acinetobacter (phylum Proteobacteria) | ^3^ | ^38^ | ^38^ | ^39^ |
| 9 | Seonamhaeicola (phylum Bacteroidetes) |  | ^40^ |  |  |
| 9 | Mesorhizobium (phylum Proteobacteria) | ^3^ |  |  |  |
| 9 | Collimonas (phylum Proteobacteria) |  | ^41^ |  |  |
| 8 | Winogradskyella (phylum Bacteroidetes) |  | ^42^ |  |  |
| 8 | Tenacibaculum (phylum Bacteroidetes) |  | ^43^ |  |  |
| 8 | Cupriavidus (phylum Proteobacteria) | ^3^ |  |  |  |
| 8 | Achromobacter (phylum Proteobacteria) | ^3^ |  |  |  |
| 7 | Thioalkalivibrio (phylum Proteobacteria) |  | ^44^ |  |  |
| 7 | Paraburkholderia (phylum Proteobacteria) |  | ^45^ |  |  |
| 7 | Mycolicibacterium (phylum Actinobacteria) |  | ^46^ |  |  |
| 7 | Haliscomenobacter (phylum Bacteroidetes) |  | ^19^ |  |  |
| 7 | Bordetella (phylum Proteobacteria)^1^ |  | ^47^ | ^47^ |  |
| 7 | Aeromonas (phylum Proteobacteria)^1^ |  | ^48^ |  |  |
| 7 | Actinomyces (phylum Actinobacteria) | ^28^ |  |  |  |
| 6 | Rhizobium (phylum Proteobacteria) | ^3^ |  |  |  |
| 6 | Ralstonia (phylum Proteobacteria) | ^3^ |  |  |  |
| 6 | Pandoraea (phylum Proteobacteria)^1,3^ |  | ^49^ |  |  |
| 6 | Mycobacterium (phylum Actinobacteria)^1^ |  | ^50^ | ^51^ |  |
| 6 | Marinomonas (phylum Proteobacteria) |  | ^52^ |  |  |
| 6 | Bosea (phylum Proteobacteria) | ^3^ |  |  |  |

1. Podschun, R., Pietsch, S., Holler, C. & Ullmann, U. Incidence of Klebsiella species in surface waters and their expression of virulence factors. *Appl Environ Microbiol* **67**, 3325-3327 (2001).

2. Podschun, R. & Ullmann, U. Klebsiella spp. as nosocomial pathogens: epidemiology, taxonomy, typing methods, and pathogenicity factors. *Clinical microbiology reviews* **11**, 589-603 (1998).

3. Salter, S.J.*, et al.* Reagent and laboratory contamination can critically impact sequence-based microbiome analyses. *BMC Biol* **12**, 87 (2014).

4. Rudolf, I.*, et al.* 16S rRNA gene-based identification of cultured bacterial flora from host-seeking Ixodes ricinus, Dermacentor reticulatus and Haemaphysalis concinna ticks, vectors of vertebrate pathogens. *Folia Microbiol (Praha)* **54**, 419-428 (2009).

5. Tenaillon, O., Skurnik, D., Picard, B. & Denamur, E. The population genetics of commensal Escherichia coli. *Nat Rev Microbiol* **8**, 207-217 (2010).

6. Lee, Y.M.*, et al.* Comparative Analysis of Lacinutrix Genomes and Their Association with Bacterial Habitat. *PLoS One* **11**, e0148889 (2016).

7. Sawabe, T.*, et al.* Pseudoalteromonas bacteriolytica sp. nov., a marine bacterium that is the causative agent of red spot disease of Laminaria japonica. *Int J Syst Bacteriol* **48 Pt 3**, 769-774 (1998).

8. Jeong, S.H., Jin, H.M., Kim, J.M. & Jeon, C.O. Arenibacter hampyeongensis sp. nov., a marine bacterium isolated from a tidal flat. *Int J Syst Evol Microbiol* **63**, 679-684 (2013).

9. Jarvis, G.N., Strompl, C., Moore, E.R. & Thiele, J.H. Isolation and characterisation of obligately anaerobic, lipolytic bacteria from the rumen of red deer. *Syst Appl Microbiol* **21**, 135-143 (1998).

10. Techtmann, S.M.*, et al.* Colwellia psychrerythraea Strains from Distant Deep Sea Basins Show Adaptation to Local Conditions. *Frontiers in Environmental Science* **4**(2016).

11. Ribeiro, T.G.*, et al.* Citrobacter europaeus sp. nov., isolated from water and human faecal samples. *Int J Syst Evol Microbiol* **67**, 170-173 (2017).

12. Cabral, J.P. Water microbiology. Bacterial pathogens and water. *Int J Environ Res Public Health* **7**, 3657-3703 (2010).

13. Wang, Y., Gao, L., Ming, H., Zhang, P. & Zhu, W. Polaribacter marinaquae sp. nov., isolated from seawater. *Int J Syst Evol Microbiol* **66**, 4594-4599 (2016).

14. Levantesi, C.*, et al.* Salmonella in surface and drinking water: Occurrence and water-mediated transmission. *Food Research International* **45**, 587-602 (2012).

15. Heredia, N. & Garcia, S. Animals as sources of food-borne pathogens: A review. *Anim Nutr* **4**, 250-255 (2018).

16. He, L.*, et al.* Streptomyces jietaisiensis sp. nov., isolated from soil in northern China. *Int J Syst Evol Microbiol* **55**, 1939-1944 (2005).

17. Seipke, R.F., Kaltenpoth, M. & Hutchings, M.I. Streptomyces as symbionts: an emerging and widespread theme? *FEMS Microbiol Rev* **36**, 862-876 (2012).

18. Arahal, D.R.*, et al.* Marinomonas blandensis sp. nov., a novel marine gammaproteobacterium. *Int J Syst Evol Microbiol* **66**, 5544-5549 (2016).

19. Daligault, H.*, et al.* Complete genome sequence of Haliscomenobacter hydrossis type strain (O). *Stand Genomic Sci* **4**, 352-360 (2011).

20. Yakimov, M.M.*, et al.* Oleispira antarctica gen. nov., sp. nov., a novel hydrocarbonoclastic marine bacterium isolated from Antarctic coastal sea water. *Int J Syst Evol Microbiol* **53**, 779-785 (2003).

21. Johansen, J.E., Nielsen, P. & Sjoholm, C. Description of Cellulophaga baltica gen. nov., sp. nov. and Cellulophaga fucicola gen. nov., sp. nov. and reclassification of [Cytophaga] lytica to Cellulophaga lytica gen. nov., comb. nov. *Int J Syst Bacteriol* **49 Pt 3**, 1231-1240 (1999).

22. Reidl, J. & Klose, K.E. Vibrio cholerae and cholera: out of the water and into the host. *FEMS Microbiology Reviews* **26**, 125-139 (2002).

23. Osunla, C.A. & Okoh, A.I. Vibrio Pathogens: A Public Health Concern in Rural Water Resources in Sub-Saharan Africa. *Int J Environ Res Public Health* **14**(2017).

24. Bridel, S.*, et al.* Comparative Genomics of Tenacibaculum dicentrarchi and "Tenacibaculum finnmarkense" Highlights Intricate Evolution of Fish-Pathogenic Species. *Genome Biol Evol* **10**, 452-457 (2018).

25. Lee, S.D. Tamlana crocina gen. nov., sp. nov., a marine bacterium of the family Flavobacteriaceae, isolated from beach sediment in Korea. *Int J Syst Evol Microbiol* **57**, 764-769 (2007).

26. Hameed, A.*, et al.* Siansivirga zeaxanthinifaciens gen. nov., sp. nov., a novel zeaxanthin-producing member of the family Flavobacteriaceae isolated from coastal seawater of Taiwan. *FEMS Microbiol Lett* **333**, 37-45 (2012).

27. Lewis, E.R. & Torres, A.G. The art of persistence-the secrets to Burkholderia chronic infections. *Pathog Dis* **74**(2016).

28. Glassing, A., Dowd, S.E., Galandiuk, S., Davis, B. & Chiodini, R.J. Inherent bacterial DNA contamination of extraction and sequencing reagents may affect interpretation of microbiota in low bacterial biomass samples. *Gut Pathog* **8**, 24 (2016).

29. Laurence, M., Hatzis, C. & Brash, D.E. Common contaminants in next-generation sequencing that hinder discovery of low-abundance microbes. *PLoS One* **9**, e97876 (2014).

30. Nedashkovskaya, O.I.*, et al.* Lacinutrix algicola sp. nov. and Lacinutrix mariniflava sp. nov., two novel marine alga-associated bacteria and emended description of the genus Lacinutrix. *Int J Syst Evol Microbiol* **58**, 2694-2698 (2008).

31. Nedashkovskaya, O.I., Suzuki, M., Vysotskii, M.V. & Mikhailov, V.V. Arenibacter troitsensis sp. nov., isolated from marine bottom sediment. *Int J Syst Evol Microbiol* **53**, 1287-1290 (2003).

32. Weon, H.Y.*, et al.* Two novel species, Lysobacter daejeonensis sp. nov. and Lysobacter yangpyeongensis sp. nov., isolated from Korean greenhouse soils. *Int J Syst Evol Microbiol* **56**, 947-951 (2006).

33. Krzysciak, W., Pluskwa, K.K., Jurczak, A. & Koscielniak, D. The pathogenicity of the Streptococcus genus. *Eur J Clin Microbiol Infect Dis* **32**, 1361-1376 (2013).

34. Becker, K., Heilmann, C. & Peters, G. Coagulase-negative staphylococci. *Clinical microbiology reviews* **27**, 870-926 (2014).

35. Tong, S.Y., Davis, J.S., Eichenberger, E., Holland, T.L. & Fowler, V.G., Jr. Staphylococcus aureus infections: epidemiology, pathophysiology, clinical manifestations, and management. *Clinical microbiology reviews* **28**, 603-661 (2015).

36. Xie, C.H. & Yokota, A. Dyella japonica gen. nov., sp. nov., a gamma-proteobacterium isolated from soil. *Int J Syst Evol Microbiol* **55**, 753-756 (2005).

37. Oda, Y., Star, B., Huisman, L.A., Gottschal, J.C. & Forney, L.J. Biogeography of the purple nonsulfur bacterium Rhodopseudomonas palustris. *Appl Environ Microbiol* **69**, 5186-5191 (2003).

38. Wong, D.*, et al.* Clinical and Pathophysiological Overview of Acinetobacter Infections: a Century of Challenges. *Clinical microbiology reviews* **30**, 409-447 (2017).

39. Sangare, A.K., Doumbo, O.K. & Raoult, D. Management and Treatment of Human Lice. *Biomed Res Int* **2016**, 8962685 (2016).

40. Park, S., Won, S.M., Park, D.S. & Yoon, J.H. Seonamhaeicola aphaedonensis gen. nov., sp. nov., a member of the family Flavobacteriaceae isolated from a tidal flat sediment. *Int J Syst Evol Microbiol* **64**, 1876-1881 (2014).

41. Hoppener-Ogawa, S.*, et al.* Collimonas arenae sp. nov. and Collimonas pratensis sp. nov., isolated from (semi-)natural grassland soils. *Int J Syst Evol Microbiol* **58**, 414-419 (2008).

42. Lee, D.H., Cho, S.J., Kim, S.M. & Lee, S.B. Winogradskyella damuponensis sp. nov., isolated from seawater. *Int J Syst Evol Microbiol* **63**, 321-326 (2013).

43. Suzuki, M., Nakagawa, Y., Harayama, S. & Yamamoto, S. Phylogenetic analysis and taxonomic study of marine Cytophaga-like bacteria: proposal for Tenacibaculum gen. nov. with Tenacibaculum maritimum comb. nov. and Tenacibaculum ovolyticum comb. nov., and description of Tenacibaculum mesophilum sp. nov. and Tenacibaculum amylolyticum sp. nov. *Int J Syst Evol Microbiol* **51**, 1639-1652 (2001).

44. Sorokin, D.Y.*, et al.* Thioalkalimicrobium aerophilum gen. nov., sp. nov. and Thioalkalimicrobium sibericum sp. nov., and Thioalkalivibrio versutus gen. nov., sp. nov., Thioalkalivibrio nitratis sp.nov., novel and Thioalkalivibrio denitrificancs sp. nov., novel obligately alkaliphilic and obligately chemolithoautotrophic sulfur-oxidizing bacteria from soda lakes. *Int J Syst Evol Microbiol* **51**, 565-580 (2001).

45. Choi, G.M. & Im, W.T. Paraburkholderia azotifigens sp. nov., a nitrogen-fixing bacterium isolated from paddy soil. *Int J Syst Evol Microbiol* **68**, 310-316 (2018).

46. Ito, T.*, et al.* Draft Genome Sequences of <span class="named-content genus-species" id="named-content-1">Mycolicibacter senuensis</span> Isolate GF74 and <span class="named-content genus-species" id="named-content-2">Mycobacterium colombiense</span> Isolates GF28 and GF76 from a Swine Farm in Japan. *Microbiology Resource Announcements* **7**(2018).

47. Hamidou Soumana, I., Linz, B. & Harvill, E.T. Environmental Origin of the Genus Bordetella. *Front Microbiol* **8**, 28 (2017).

48. Igbinosa, I.H., Igumbor, E.U., Aghdasi, F., Tom, M. & Okoh, A.I. Emerging Aeromonas species infections and their significance in public health. *ScientificWorldJournal* **2012**, 625023 (2012).

49. Kostygov, A.Y.*, et al.* Genome of Ca. Pandoraea novymonadis, an Endosymbiotic Bacterium of the Trypanosomatid Novymonas esmeraldas. *Front Microbiol* **8**, 1940 (2017).

50. Santos, N.*, et al.* Widespread Environmental Contamination with Mycobacterium tuberculosis Complex Revealed by a Molecular Detection Protocol. *PLoS One* **10**, e0142079 (2015).

51. Rahman, S.A.*, et al.* Comparative analyses of nonpathogenic, opportunistic, and totally pathogenic mycobacteria reveal genomic and biochemical variabilities and highlight the survival attributes of Mycobacterium tuberculosis. *MBio* **5**, e02020 (2014).

52. Romanenko, L.A., Uchino, M., Mikhailov, V.V., Zhukova, N.V. & Uchimura, T. Marinomonas primoryensis sp. nov., a novel psychrophile isolated from coastal sea-ice in the Sea of Japan. *Int J Syst Evol Microbiol* **53**, 829-832 (2003).
