# Supplementary material for: High-throughput metagenome analysis of the Sarcoptes scabiei internal microbiota and in-situ identification of intestinal Streptomyces sp
Source: Sci Rep. 2019 Aug 13;9:11744. doi: 10.1038/s41598-019-47892-0 (PMC6692375; doi:10.1038/s41598-019-47892-0)

High-throughput metagenome analysis of the *Sarcoptes scabiei* internal microbiota and *in-situ* identification of intestinal *Streptomyces sp*.

[1]Pearl M. Swe+, [2]Martha Zakrzewski+, [1]Rebecca Waddell, [1]Kadaba S. Sriprakash and [1]Katja Fischer

S2_Fig 1. Krona plots of bacterial abundance in the metagenomes of (a) adult female scabies mites and (b) scabies eggs. Sequence data were annotated using Kraken and visualized using Krona softwares.

(a)

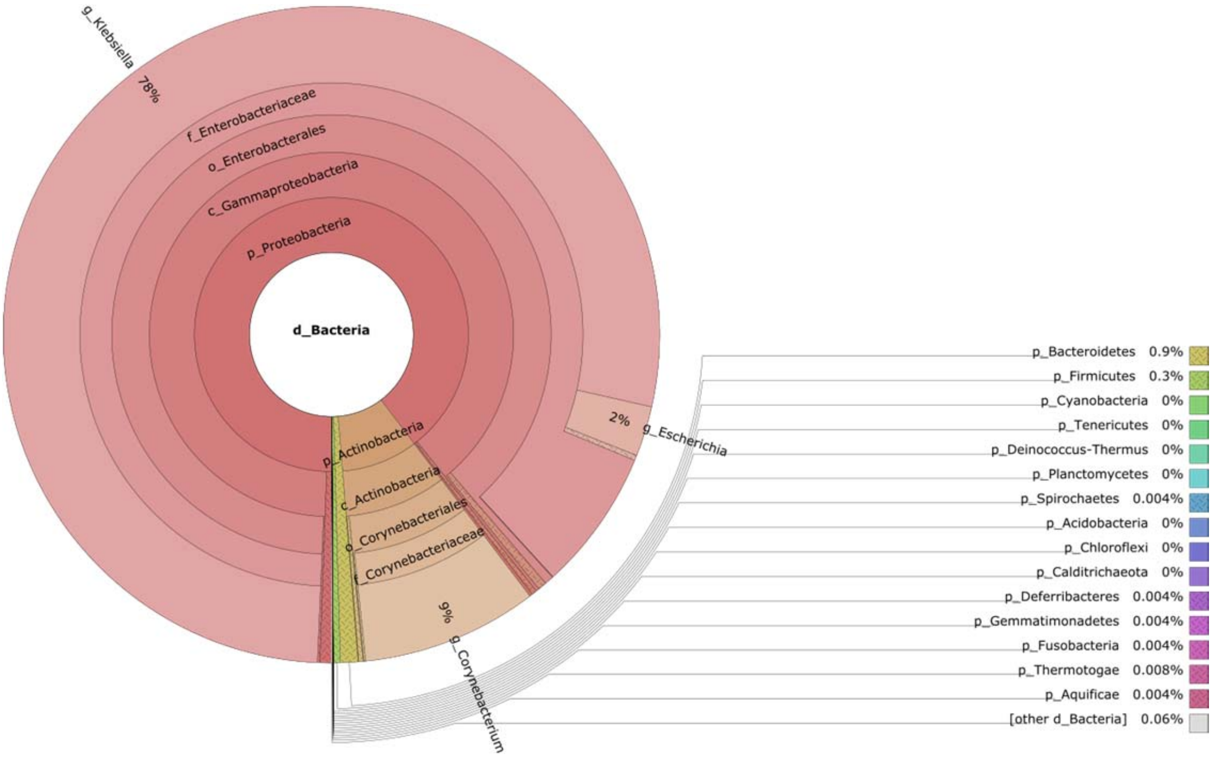

(b)

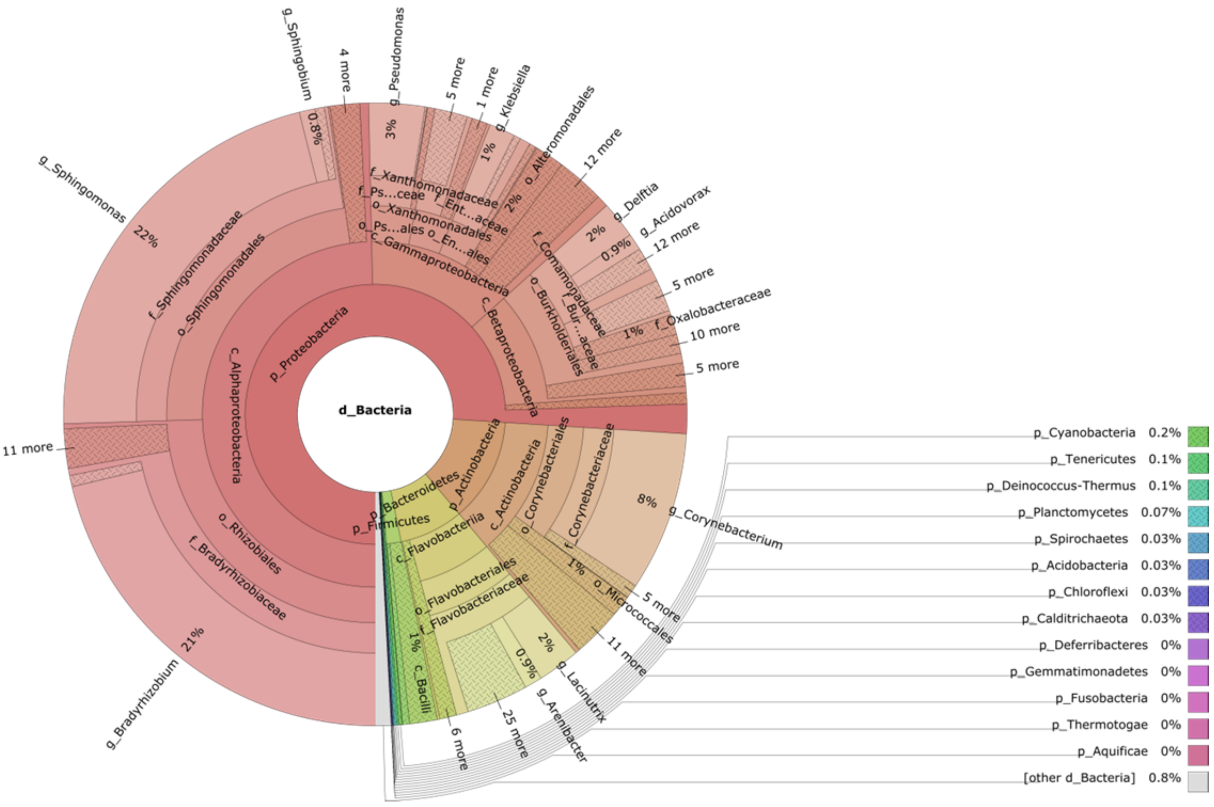

Supplement: Supplementary file 2 — Supplementary Dataset 2 [file 41598_2019_47892_MOESM2_ESM.pdf]
